# Mediator Med23 Regulates Adult Hippocampal Neurogenesis

**DOI:** 10.3389/fcell.2020.00699

**Published:** 2020-07-29

**Authors:** Guo-Yan Chen, Shuai Zhang, Chong-Hui Li, Cong-Cong Qi, Ya-Zhou Wang, Jia-Yin Chen, Gang Wang, Yu-Qiang Ding, Chang-Jun Su

**Affiliations:** ^1^Department of Neurology, Tangdu Hospital, Air Force Medical University (Fourth Military Medical University), Xi’an, China; ^2^Key Laboratory of Arrhythmias, Ministry of Education of China, East Hospital, and Department of Anatomy and Neurobiology, Tongji University School of Medicine, Shanghai, China; ^3^State Key Laboratory of Cell Biology, CAS Center for Excellence in Molecular Cell Science, Shanghai Institute of Biochemistry and Cell Biology, Chinese Academy of Sciences, University of Chinese Academy of Sciences, Shanghai, China; ^4^School of Life Sciences and Technology, ShanghaiTech University, Shanghai, China; ^5^State Key Laboratory of Medical Neurobiology and MOE Frontiers Center for Brain Science, Institute of Brain Science, and Department of Laboratory Animal Science, Fudan University, Shanghai, China; ^6^Department of Neurobiology, Institute of Neurosciences, School of Basic Medicine, Fourth Military Medical University, Xi’an, China; ^7^School of Life Sciences, Fudan University, Shanghai, China

**Keywords:** Mediator complex 23, hippocampus, proliferation, cell cycle, adult neural stem cells

## Abstract

Mammalian Mediator (Med) is a key regulator of gene expression by linking transcription factors to RNA polymerase II (Pol II) transcription machineries. The Mediator subunit 23 (Med23) is a member of the conserved Med protein complex and plays essential roles in diverse biological processes including adipogenesis, carcinogenesis, osteoblast differentiation, and T-cell activation. However, its potential functions in the nervous system remain unknown. We report here that Med23 is required for adult hippocampal neurogenesis in mouse. Deletion of Med23 in adult hippocampal neural stem cells (NSCs) was achieved in Nestin-Cre^ER^:Med23^flox/flox^ mice by oral administration of tamoxifen. We found an increased number of proliferating NSCs shown by pulse BrdU-labeling and immunostaining of MCM2 and Ki67, which is possibly due to a reduction in cell cycle length, with unchanged GFAP^+^/Sox2^+^ NSCs and Tbr2^+^ progenitors. On the other hand, neuroblasts and immature neurons indicated by NeuroD and DCX were decreased in number in the dentate gyrus (DG) of Med23-deficient mice. In addition, these mice also displayed defective dendritic morphogenesis, as well as a deficiency in spatial and contextual fear memory. Gene ontology (GO) analysis of hippocampal NSCs revealed an enrichment in genes involved in cell proliferation, Pol II-associated transcription, Notch signaling pathway and apoptosis. These results demonstrate that Med23 plays roles in regulating adult brain neurogenesis and functions.

## Introduction

The mammalian Mediator complex is an evolutionarily conserved multi-protein complex, which functions as a key transcriptional cofactor by forming a link between sequence-specific transcription factors and the RNA polymerase II (Pol II)-associated basal transcription machinery. In this context, it may be required for the transcription of thousands of protein-coding genes (Lewis and Reinberg, 2003; Malik and Roeder, 2005; Hentges, 2011). The Mediator complex contains up to 30 proteins, and given its fundamental role in gene transcription, inactivation of individual genes may lead to global gene expression defects. However, based on data showing that mutation of different Mediator complex genes in the same organism leads to different phenotypes, individual Mediator complex proteins have unique biological functions (Bourbon et al., 2004; Malik and Roeder, 2010; Hentges, 2011).

The Mediator subunit 23 (Med23, also known as sur2) was originally identified as a genetic suppressor of activated let-60 ras mutation in *Caenorhabditis elegans* (Singh and Han, 1995). Med23 functions in the mitogen-activated protein kinase (MAPK) signaling pathway by interacting with the ternary complex factor Elk1, and data from embryonic fibroblasts have indicated that MAPK-activated interaction of Med23-Elk1 is required for hormone-induced adipogenesis through controlling transcription of immediate early gene Egr2 (Stevens et al., 2002; Wang et al., 2005, 2009). Important roles of Med23 have also been reported in other biological processes including osteoblast differentiation (Liu et al., 2016), myogenesis (Yin et al., 2012), lung carcinogenesis (Yang et al., 2012; Yao et al., 2015), glucose and lipid metabolism (Chu et al., 2014), and T-cell activation (Sun et al., 2014).

Our previous study has shown that Med23-deficient embryonic stem cells display enhanced neural differentiation (Zhu et al., 2015). However, the roles of Med23 in the nervous system remain unknown. Importantly, a missense mutation of Med23 has been reported in patients with intellectual disability and dysregulation of expression of immediate early gene JUN and FOS (Hashimoto et al., 2011; Trehan et al., 2015). In this study, we focused on adult hippocampal neurogenesis in order to explore possible roles of Med23 within the brain.

Adult neurogenesis in the mammalian brain has received increased attention in recent years, especially because of its potential roles in neurological and psychiatric disorders. The process of adult neurogenesis includes the proliferation and differentiation of neural stem cells (NSCs), neuronal survival and migration, and integration of newborn neurons into existing circuits (Zhao et al., 2008; Ming and Song, 2011). The subgranular zone (SGZ) in the dentate gyrus (DG) of the hippocampus is one of the active neurogenic niches of the adult brain (Morales and Mira, 2019). Impaired SGZ neurogenesis is associated with defective spatial learning and memory, and retrieval of contextual fear memory in mice (Zhang et al., 2013, 2014; Lieberwirth et al., 2016). It has also been reported that enhanced hippocampal neurogenesis is involved in mediating the antidepressant effects of fluoxetine and cognition in Alzheimer’s mouse model (Santarelli et al., 2003; Choi et al., 2018).

Here, we provide evidence that Med23 is involved in the regulation of adult hippocampal neurogenesis in mouse. Med23-deficient NSCs in the SGZ display faster self-renewal activity possibly by reducing cell cycle length, but neuroblasts, immature and mature new-born neurons are reduced. In addition, inducible Med23 conditional knockout (CKO) mice show defects in spatial and contextual fear memory. Gene ontology (GO) analysis of Med23-deficient NSCs reveals an enrichment in genes involved in cell proliferation, Pol II-associated transcription, Notch signaling pathway and apoptosis. Taken together, these results demonstrate that Med23 is an important regulator of adult brain functions.

## Materials and Methods

### Animals

To investigate the role of Med23 in adult hippocampal neurogenesis, we first generated Nestin-Cre^ER^:Med23^flox/+^ mice by crossing Nestin-Cre^ER^ (Imayoshi et al., 2008) with Med23^flox/flox^ mice with two LoxP sites flanking exons 5–7 of Med23 allele as described in our previous study (Chu et al., 2014). Nestin-Cre^ER^:Med23^flox/+^ mice were further crossed with Med23^flox/flox^ mice to obtain Nestin-Cre^ER^:Med23^flox/flox^ (Med23 CKO) mice. Littermates with other genotypes (i.e., Med23^flox/+^ and Med23^flox/flox^) were used as controls, because they did not exhibit any detectable alterations in the SGZ (see below). To visualize Med23-deficient NSCs and its progeny *in vivo* using Rosa26-stop-YFP mice (Romer et al., 2011), Nestin-Cre^ER^:Med23^flox/flox^:Rosa26-stop-YFP (referred to as Med23 CKO:Rosa-YFP) mice were generated by crossing Nestin-Cre^ER^:Rosa26-stop-YFP:Med23^flox/+^ mice with Med23^flox/+^ mice; in this set of experiments littermates with the genotype of Nestin-Cre^ER^:Rosa26-YFP:Med23^+/+^ were used as controls, because they did not show any detectable alterations in the SGZ either. All animal experiments were carried out under the protocols approved by the Animal Care and Use Committees of Tongji University School of Medicine, China.

### Tamoxifen Administration

To activate Cre-mediated recombination, Tamoxifen (TAM; 200 mg/kg; Sigma) dissolved in corn oil solution (Sigma) was administered once daily by oral gavage for 5 consecutive days in adult (2 months) Med23 CKO mice. Littermate control mice received the same treatment. TM-treated mice were allowed to recover 3–4 weeks and then used in most experiments. In the experiment of hippocampal NSC culture, the TAM treatment were done at the age of one month.

### BrdU and EdU Administration

To observe the survival of newborn neurons, mice were treated with TAM as described above, followed by intraperitoneal (i.p.) injection of 5-bromo-2′-deoxyuridine (BrdU; 100 mg/kg; Sigma) the day after, daily for 3 consecutive days, and sacrificed 21 days later. To observe proliferation of NSCs in the SGZ, mice were injected with BrdU (50 mg/kg) four times with a 2 h interval in-between injections were sacrificed 2 h after the last injection. To analyze cell cycle length of NSCs, mice received a single injection of BrdU (50 mg/kg), and were sacrificed 30 min later for double immunostaining of BrdU and Ki67 as described previously (Qu et al., 2013).

To explore the length of the S-phase of NSCs, mice were first injected with 57.5 mg/kg of 5-ethynyl-2′-deoxyuridine (EdU; Life Technologies), followed by another single injection of 42.5 mg/kg BrdU 3 h later. Mice were sacrificed 45 min later for double staining of BrdU and EdU as described previously (Brandt et al., 2012).

### Immunostaining of BrdU, EdU, and Ki67

For BrdU detection, sections were incubated in 2N HCl for 25 min at 37°C prior to neutralization with 0.01 M sodium borate (pH 8.5) for 10 min. Sections were washed in phosphate-buffered saline (PBS) and incubated with mouse anti-BrdU (1:400; Calbiochem) at 4°C overnight. Then sections were incubated with biotinylated horse anti-mouse (1:500; Vector) for 3 h and finally with Cy3-conjugated streptavidin (1:1000; Sigma) for 1 h at room temperature (RT).

For double staining of BrdU and EdU, sections were processed for detection of BrdU first as described above, followed by EdU staining as follows: incubation of sections for 3 h at RT in PBS containing 1 mM CuSO4, 50 mM ascorbic acid and 10 μM fluorescent azide 488 (Salic and Mitchison, 2008).

For double staining of BrdU and Ki67, HCl/sodium borate-pretreated sections were incubated with a mixture of rat anti-BrdU (1:1000; Accurate) and rabbit anti-Ki67 (1:400; Leica), then with biotinylated horse anti-rat IgG (1:500; Vector) for 3 h, and finally with a mixture of Cy3-conjugated streptavidin (1:1000; Sigma) and 488-donkey anti-rabbit (1:500; Invitrogen) for 1 h at RT.

### Immunostaining, *in situ* Hybridization and TUNEL Staining

Under deep anesthesia with sodium pentobarbital (Merck), mice were transcardially perfused with 0.1 M PBS (pH 7.4) followed by 4% paraformaldehyde (PFA) in PBS. After cryoprotection with 30% sucrose in PBS, brains were cut into 30-μm-thick coronal sections on a cryostat, which were then treated with 0.01 M citrate buffer (pH 6.0) at 95°C for 7 min before incubating with the following primary antibodies for single or double immunostaining: rabbit anti-Ki67 (1:400; Leica), mouse anti-MCM2 (1:800; BD Pharmingen), goat anti-Sox2 (1:200; Santa Cruz), rabbit anti-glial fibrillary acidic protein (GFAP; 1:1000; DAKO), goat anti-doublecortin (DCX; 1:400; Santa Cruz), goat anti-NeuroD (1:400; Santa Cruz), rabbit anti-GFP (1:1000; Life Tech), mouse anti-NeuN (1:1000; Millipore) or rabbit anti-cleaved caspse3 (1:1000; Abcam). Sections were washed in PBS and incubated with appropriate secondary antibodies: biotinylated horse anti-goat IgG (1:500; Vector), 488-donkey anti-rabbit (1:500; Invitrogen), biotinylated goat anti-rabbit (1:500; Vector), biotinylated horse anti-mouse (1:500; Vector) or 488-donkey anti-rabbit (1:500; Invitrogen) at RT for 3 h. For biotinylated secondary antibodies, sections were washed in PBS and incubated with Cy3-conjugated streptavidin (1:1000; Sigma) for 1 h at RT. In addition, *in situ* hybridization of Tbr2 was performed in brain slice as described previously (Pruski et al., 2019), the sequence of primers for making RNA probe of Tbr2 were: Forward, 5′-TTATCAGAGGAAGATGGCAGC-3′; Reverse, 5′-AGAGCCCACTGTTAACTCAAGG-3′. TUNEL staining was performed in cultured NSCs and brain slice as described previously (Ding et al., 2003; Yang et al., 2018; Pruski et al., 2019).

### Western Blotting

Hippocampal tissues were homogenized in RIPA lysis buffer as described previously (Yang et al., 2018). After SDS-PAGE and protein transfer, membranes were incubated with following primary antibodies: rabbit anti-DRIP130 (1:2000; Abcam) and mouse anti-β-actin (1:5000; Sigma) overnight at 4°C, followed by incubation with HRP-conjugated anti-rabbit or anti-mouse IgG (1:2000; Proteintech) for 1 h at RT. Antibodies were visualized using an ECL kit (Thermo Fisher Scientific).

### Stereotactic Injections

After anesthetizing with 10% chloral hydrate (0.4 ml/100 g body weight), mice were stereotactically injected with 1 μl of retrovirus expressing GFP (pRov-U6-shRNA-EF1a-EGFP) into the DG. Coordinates from the Bregma were (in mm): −1.94 anterior/posterior ± 1.25 medial/lateral, and −2.0 dorsal/ventral from the dura mater. Twenty-one days later, animals were perfused as described above. Dendritic lengths and branch points of EGFP-labeled newborn neurons in the SGZ were traced with ImageJ and analyzed.

### Hippocampal NSC Culture

Primary NSC cultures were prepared from the hippocampus of 2-month-old Med23 CKO and littermate control mice as described previously with some modifications (Brewer and Torricelli, 2007). Briefly, under anesthesia with chloral hydrate, the hippocampus was removed and dissected in HABG, which contained B27 (1×; Invitrogen), Glutamax (0.25×; Invitrogen), and HA (1×; GIBCO). Hippocampal tissues were digested in 0.025% Trypsin-EDTA (1×; GIBCO) for 15 min at 37°C, then DMEM + 10% FBS was added to terminate the reaction before centrifuging at 800 rpm for 15 min, and the tissue was finally dissociated into a single-cell suspension by mechanical disruption. The single-cell suspension was grown in Neurobasal-A Medium (1×; GIBCO), supplemented with B27-A (1×; Invitrogen), L-glutamine (1×; Invitrogen), FGF (20 ng/ml; Invitrogen), EGF (20 ng/ml; Invitrogen), N2 (1×; Invitrogen), and gentamicin (0.01 mg/ml; Invitrogen). The desired cells were incubated at 37°C in 5% CO_2_ for 8 days until primary neurospheres were formed. Primary neurospheres were mechanically dissociated into single cells and incubated for 5–6 days to allow secondary neurosphere formation. The sizes through measuring the length of primary and secondary neurospheres were recorded under a microscope (Nikon). Finally, primary neurospheres were harvested for quantitative real-time RT-PCR (qRT-PCR) (see below).

To evaluate the proportion of dividing cells in the neurospheres, primary neurospheres were treated with 10 μM BrdU for 4 h and then subjected to immunostaining of BrdU as mentioned above.

### RNA Extraction and qRT-PCR

Analyses were conducted with RNA extracts from primary neurospheres from Med23 CKO and control mice, harvested after 8 days of incubation. Total RNA was extracted as per Trizol (Life Technologies) protocol. cDNA was generated with iScript^TM^ cDNA Synthesis Kit (Bio-Rad). qRT-PCR was performed using the ABI Prism 7500 Sequence Detector System. Primer sequences are as follows: β-actin (Forward: 5′-GGCTGTATTCCCCTC CATCG-3′; Reverse: 5′-CCAGTTGGTAACAATGCCATGT-3′), Med23 (Forward: 5′-AGGAGTGGATTACAAGGGTG-3′; Reverse: 5′-TAGGCAGGCATTTCGTTC-3′).

### RNA-Sequencing and Data Analysis

An RNA-sequencing (RNA-seq) library was prepared from 8-day cultured primary neurospheres using NEBNext^®^ Ultra^TM^ RNA Library Prep Kit for Illumina (E7530) following the manufacturer’s instructions, and all libraries were sequenced using the Illumina HiSeq platform. Raw.fastq files were analyzed using FastQC, and adapter removal was performed using cutadapt1.12. Reads were aligned to mouse genome assembly mm 10 using Tophat (Trapnell et al., 2009) with default parameters. Differential expression analysis was performed using Cuffdiff (Trapnell et al., 2012) and FPKM was calculated. GO analysis of differentially expressed genes was carried out using DAVID online tools^1^.

### Behavioral Tests

Adult male mice (3–4 months old) were used in behavioral tests, and in the other experiments mice with either of sex were used. The experiments were performed during 9:00 a.m. to 5:00 p.m., and animal behaviors were videotaped. Mice were habituated in the test room for over 30 min before behavioral experiments. Ethanol (75%) was used to clean arenas and objects between trials to remove excrements and odors for the following behavioral test (except forced swimming and tail suspension test). All behavioral tests were performed by a trained person who was blind to the genotypes.

#### Morris Water Maze

The Morris water maze was used to examine spatial memory (Vorhees and Williams, 2006). The apparatus contained two parts: a circle tank (120-cm diameter) and a hidden platform (10-cm diameter) submerged 1–2 cm below the surface of the opaque water (24–26°C). Mice movements were monitored and analyzed using EthoVision (8.0). In the spatial learning phase, mice were trained for four trials per day for 6 days at intertrial intervals of 40–50 min to discover the escape platform. The escape latencies of four trials per day were averaged for each animal. To test spatial memory, the hidden platform was removed and mice were subjected to retention tests 1 day after the spatial learning task. Memory retrieval was measured by quantifying the time spent in the target quadrant, the time taken to first cross the platform location, and the number of platform location crossings in a 1-min trial. Swim velocity was also measured during both the spatial learning and memory retrieval tasks.

#### Contextual Fear Conditioning

This test was used to assess contextual fear memory in mice, performed as described previously (Dai et al., 2008; Hu et al., 2014). In brief, mice freely explored the box for 10 min before the test, prior to being placed back in their home cage. About 1 h later, mice were placed in the box and given 5 foot shocks (1.2 mA, 2 s duration) at 2-min intervals to learning contextual fear memory. To study retrieval of contextual fear memory, mice were placed in the conditioned fear context for 11 min to observe the percentage of freezing without any foot shock at 30 min, on day 1 and day 7.

#### Novel Object Recognition Test

This test was conducted in a black sound-proof chamber, containing a Plexiglas box (25 × 25 × 25 cm) and camera. The task procedure was described previously (Wang et al., 2007; Botton et al., 2010). In brief, the procedure consisted of three phases: habituation, familiarization, and test phase. In the habituation phase, the mouse was placed into the box without any object and allowed to explore for 10 min on day 1. During familiarization, two identical sample objects (A + A) were placed into opposite corners of the box approximately 8 cm from the walls. A single mouse was placed into the box facing away from the objects and allowed to explore for 10 min on day 2. During the test phase, mouse was returned to the box which contained the location of novel (B) versus familiar object (A) about 1 h later, and allowed to explore for 5 min. Exploration was defined by directing the nose to the object at a distance of no more than 2 cm and/or touching the object with the nose or forepaw. During the test phase, a recognition index for each mouse was expressed by TN/(TN + TF) ratio (TF = time spent exploring familiar object; TN = time spent exploring novel object).

### Cell Count and Statistical Analysis

Positive cells were counted in every 7th sections in the DG from anterior to posterior, expressed as count per mm^3^, by a trained observer who was blind to genotypes. All samples showed normal distribution examined by OriginPro9.1, and unpaired Student’s *t*-test was performed using GraphPad Prism 5. Date are expressed as mean ± SEM, and *p*-values < 0.05 were considered to be statistically significant.

## Results

### Increased Number of Proliferating NSCs in Med23 CKO Mice

**FIGURE 1:**
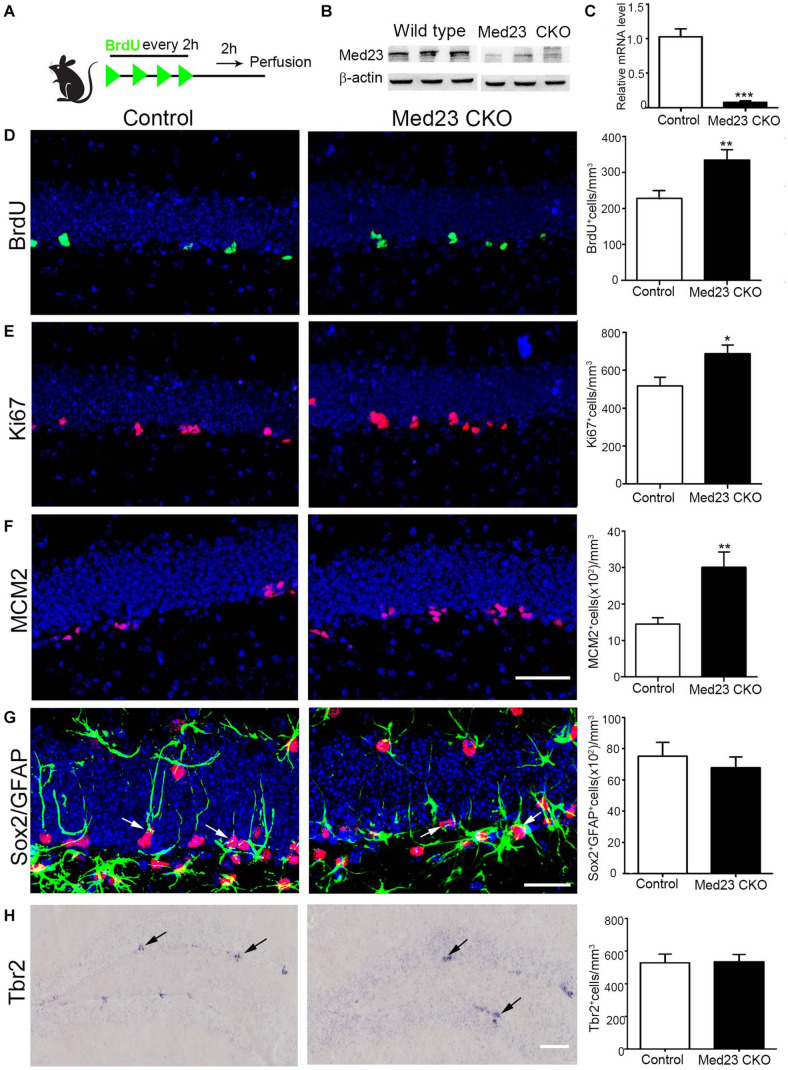
Increased proliferation of Med23-deficient hippocampal NSCs *in vivo*. **(A)** Schematic representation of BrdU injection. **(B)** Western blot of hippocampal tissues of Med23 CKO mice relative to wild type littermates. *N* = 3 for each. **(C)** qPCR data showing the Med23 mRNA levels in the primary neurospheres of control and Med23 CKO. Neurospheres from six control and three Med23CKO mice was included. **(D)** Distribution and quantification of BrdU^+^ cells in the SGZ of control and Med23 CKO. Seven control mice and six Med23 CKO mice were included. **(E)** Distribution and quantification of Ki67^+^ cells in the SGZ of control and Med23 CKO mice. *N* = 8 mice in control and seven mice in Med23 CKO groups. **(F)** Distribution and quantification of MCM2^+^ cells in the SGZ of control and Med23 CKO mice. *N* = 5 mice in each group. Scale bar = 50 μm **(D–F)**. **(G)** Distribution and quantification of Sox2^+^/GFAP^+^ cells (arrows) in the SGZ of control and Med23 CKO. *N* = 5 mice in control and six mice Med23 CKO groups. Scale bar = 25 μm. **(H)** Distribution and quantification of Tbr2^+^ cells (arrows) in the SGZ of control and Med23 CKO mice. *N* = 4 mice in each group. Scale bar = 100 μm. Data are plotted as the mean ± SEM. Student *t*-test, *p < 0.05, **p < 0.01, ***p < 0.001.

To examine whether hippocampal NSCs are affected by the inactivation of Med23, we first performed BrdU pulse-labeling (Figure 1A) and quantified BrdU^+^ cells in the SGZ as reported in our previous study (Song et al., 2016). Deletion of Med23 was confirmed by western blot of hippocampal tissues of Med23 CKO mice (Figure 1B), and by qRT-PCR analysis of Med23 transcript of neurospheres prepared from Med23 CKO mice (Figure 1C). Quantification of BrdU^+^ cells in the DG showed a significant increase in Med23 CKO mice compared with controls (Figure 1D). In addition, cell cycle markers Ki67 (Figure 1E) and MCM2 (Figure 1F) were also significantly increased in the SGZ of Med23 CKO mice compared with controls. We next examined the population of GFAP^+^/Sox2^+^ cells, which represent quiescent and active NSCs, and found no significant changes between control and Med23 CKO mice (Figure 1G). The proliferating cells in the SGZ also include progenitors that express Tbr2 (Kalamakis et al., 2019; Morales and Mira, 2019) and there was no obvious alteration in the number of Tbr2^+^ cells in the SGZ (Figure 1H). Taken together, although the whole population of NSCs is not changed the active NSCs is likely to be increased in the SGZ of adult Med23 CKO mice.

**FIGURE 2:**
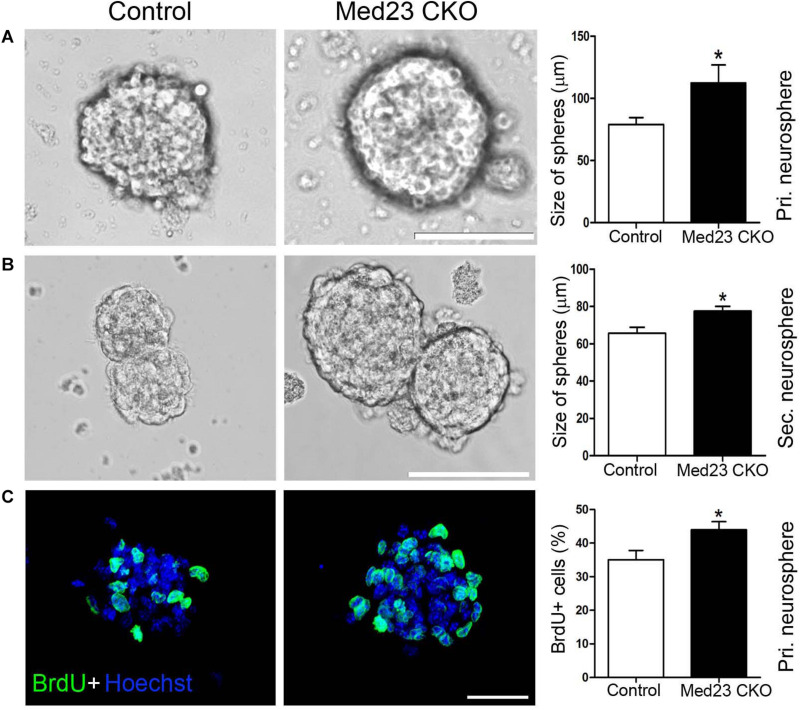
Increased proliferation of Med23-deficient NSCs *in vitro.* **(A,B)** Primary and secondary neurospheres cultured from NSCs of control and Med23 CKO mice. About 11 primary neurospheres were obtained from each mice, and a total of eight mice were used in each group. For the analysis of secondary neurospheres, eight neurospheres were included from each mouse and eight mice were used in each group. Scale bars = 100 μm. **(C)** Immunostaining of BrdU in primary neurospheres from control and Med23 CKO mice, and comparison of percentages of BrdU^+^ cells in the total cell population. About six neurospheres were included from each mouse, and five mice were used in each group. Scale bar = 25 μm. Data are plotted as the mean ± SEM. Student *t*-test, *p < 0.05.

To further confirm this observation, we isolated hippocampal NSCs from Med23 CKO and control mice and cultured them for 8 days until primary neurospheres were formed. The size of primary neurospheres was significantly increased in cultures from Med23 CKO mice relative to controls (Figure 2A). Next, the primary neurospheres were dissociated into single cells and cultured to form secondary neurosphere. The size of secondary neurospheres was also larger in Med23-deficient NSCs than in control NSCs (Figure 2B). Primary neurospheres were treated with BrdU for 4 h and the proliferation of cultured NSCs was monitored by BrdU-labeling. The percentage of BrdU^+^ cells in the total cell population was significantly increased in Med23-deficient NSCs compared with control cells (Figure 2C). Taken together, these results suggest that the self-renewal of hippocampal NSCs is enhanced in the absence of Med23.

### Reduced Cell Cycle Length of NSCs in Med23 CKO Mice

**FIGURE 3:**
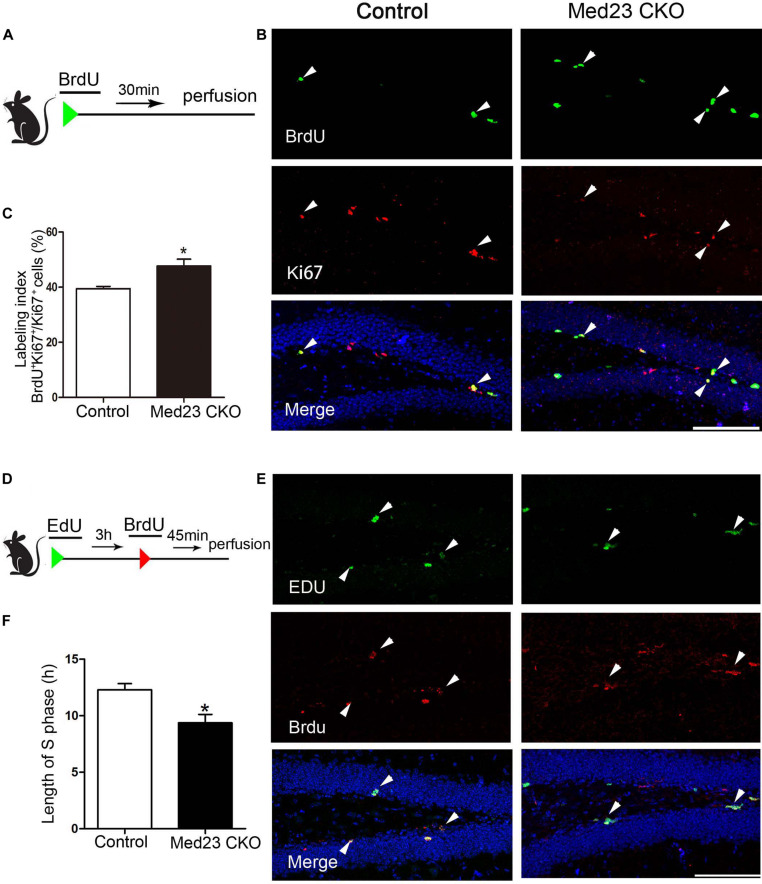
Loss of Med23 affects cell cycle progression of NSCs in the SGZ of adult mice. **(A)** Schematic representation of BrdU injection. **(B)** Double immunostaining of BrdU (green) and Ki67 (red) in BrdU-injected mice. Triangles point to BrdU^+^/Ki67^+^ cells. Hoechst (blue) was used for counterstaining. Scale bar = 100 μm. **(C)** Percentage of BrdU^+^/Ki67^+^ cells in the total of Ki67^+^ cells (labeling index) is significantly increased in Med23 CKO mice compared with controls. *N* = 5 mice in control and 4 in Med23 CKO groups. **(D)** Schematic representation of EdU and BrdU injection. **(E)** Double staining of EdU (green) and BrdU (red) in the SGZ. Triangles point to BrdU^+^/EdU^+^ cells. Hoechst (blue) was used for counterstaining. Scale bar = 100 μm. **(F)** The length of S-phase of NSCs is shorter in Med23 CKO than controls. *N* = 3 in control and 4 in Med23 CKO groups. Data are plotted as the mean ± SEM. Student *t*-test, *p < 0.05.

There are several possibilities that may underlie the phenotype of increased NSC proliferation, such as enhanced cell cycle entry via recruitment of previously quiescent stem cells, reduced cell cycle exit, or modification of cell cycle length (Fischer et al., 2014). To investigate the increased NSC proliferation in more detail, we first performed double immunostaining of BrdU and Ki67 to estimate cell cycle length. Following BrdU injection (Figure 3A), we calculated the labeling index by the ratio of BrdU^+^/Ki67^+^ cells in the total of Ki67^+^ cells as described previously (Chenn and Walsh, 2002; Qu et al., 2013). This labeling index provides an estimation of cell cycle length, in that a higher index represents a shorter cell cycle and vice versa. The index was 47.7 and 39.5% in Med23 CKO and control mice, respectively, showing that cell cycle length of hippocampal NSCs is shortened in the absence of Med23 (Figures 3B,C). As the shortened cell cycle length of NSCs may be due to changes of S-phase in relation to the total cell cycle length, we next injected EdU and BrdU in a 3-h interval (Figure 3D) to estimate S-phase by calculation of 3(h) × EdU^+^/EdU^+^BrdU^–^ cells (Brandt et al., 2012). It showed that the length of S phase in hippocampal NSCs was 9.37 h in Med23 CKO mice, thus being significantly shorter than in controls (12.29 h) (Figures 3E,F). These results suggest that the reduction of cell cycle length may contribute to the increased proliferation of NSCs in the SGZ of Med23 CKO mice.

### Reduction of Neuroblasts, and Immature and Mature Newborn Neurons in the Hippocampus of Med23 CKO Mice

**FIGURE 4:**
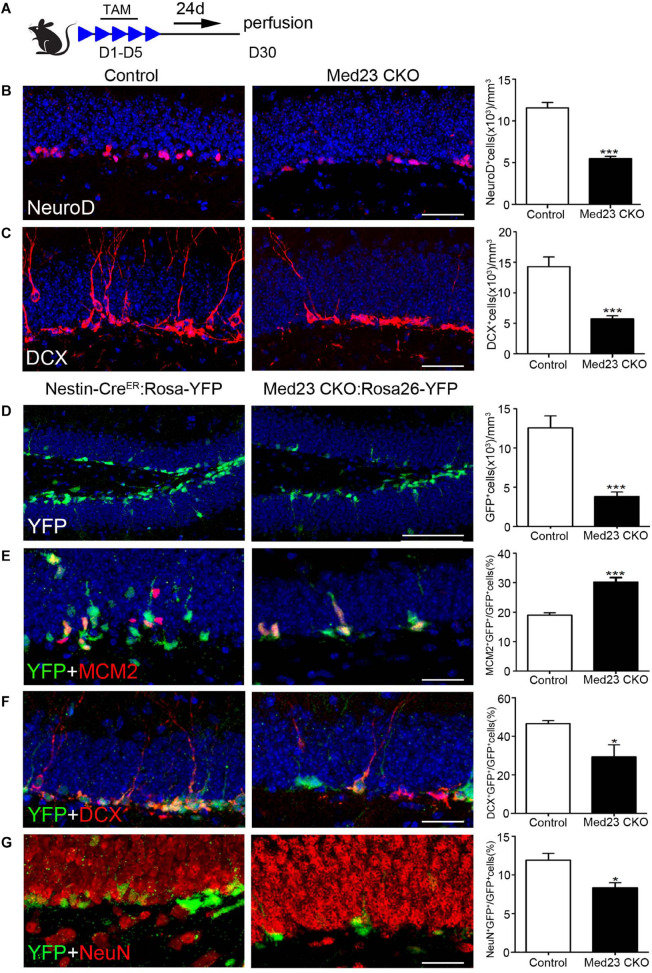
Med23 deficiency results in a reduction of neuroblasts and immature newborn neurons in the SGZ. **(A)** Schematic representation of TAM treatment. **(B,C)** Distribution and quantification of NeuroD^+^ and DCX^+^ cells in the SGZ of Med23 CKO mice compared with controls. *N* = 6 in control and 4 in Med23 CKO groups **(B)** and *n* = 5 in each group **(C)**. **(D–G)** Distribution and quantification of YFP-expressing cells (green, **D**), and its combination with MCM2 (red, **E**), DCX (red, **F**), and NeuN (red, **G**) in the SGZ of Med23 CKO:Rosa26-YFP mice and control Nestin-Cre^ER^:Rosa26-YFP with Med23^+/+^ genotype. *N* = 5 mice in each group **(D)**; *n* = 4 mice in control and 3 mice in Med23 CKO groups **(E–G)**. Scale bars = 25 μm. Data are plotted as the mean ± SEM. Student *t*-test, *p < 0.05, ***p < 0.001.

Given our finding of the increased proliferation of Med23 CKO NSCs both *in vivo* and *in vitro*, we were prompted to examine how neurogenesis is changed in Med23 CKO mice by the examination of molecular markers expressed in different stages of hippocampal neurogenesis (Duan et al., 2008; Zhao et al., 2008; Ming and Song, 2011). TAM was administered at the age of 2 months and sacrificed 4 weeks after the first TAM treatment (Figure 4A). NeuroD mainly expressed by neuroblasts and there was a significant reduction in NeuroD^+^ cells in Med23 CKO mice compared with controls (Figure 4B). In addition, DCX expressed by neuroblasts and immature neurons also displayed a reduction in cell number in Med23 CKO mice (Figure 4C). Thus, although the proliferation of hippocampal NSCs is enhanced, differentiating newborn neurons are reduced in the hippocampus of Med23 CKO mice.

To further confirm this, we generated Med23 CKO:Rosa26-YFP mice, in which YFP is expressed in Nestin^+^ NSCs and their progeny, including differentiating newborn and mature neurons. We found that YFP^+^ cells were significantly decreased in number in the SGZ of Med23 CKO:Rosa26-YFP mice relative to Nestin-Cre^ER^:Rosa26-YFP mice (Figure 4D); the Nestin-Cre^ER^:Rosa26-YFP mice with Med23^+/+^ genotype showed no obvious changes in the number of BrdU^+^, Ki67^+^, MCM2^+^, or DCX^+^ cells relative to control mice and used as control mice here. However, the percentage of cells expressing YFP^+^/MCM2^+^ in YFP^+^ cells was increased in CKO mice relative to controls (Figure 4E), which is consistent with the increase of NSCs as described above. Thus, the decrease in YFP^+^ cell number is likely caused by a reduction in the number of differentiating and/or mature newborn neurons. Indeed, the percentage of YFP^+^/DCX^+^ cells in YFP^+^ cells was reduced in Med23 CKO mice compared with controls (Figure 4F). To explore how many of the YFP^+^ cells were differentiated neurons, we performed NeuN immunostaining. It showed that some YFP^+^ cells expressed NeuN in both control and CKO mice, but it is percentage of YFP^+^/NeuN^+^ cells in YFP^+^ cells was significantly reduced in Med23 CKO mice (Figure 4G). Noted that no significant differences in the proportion of YFP^–^/MCM2^+^ cells in the total of MCM2^+^ or YFP^–^/DCX^+^ cells in the population of DCX^+^ cells were detected between the two groups. There results demonstrate that the neurogenesis is impaired in the SGZ of Med23 CKO mice.

**FIGURE 5:**
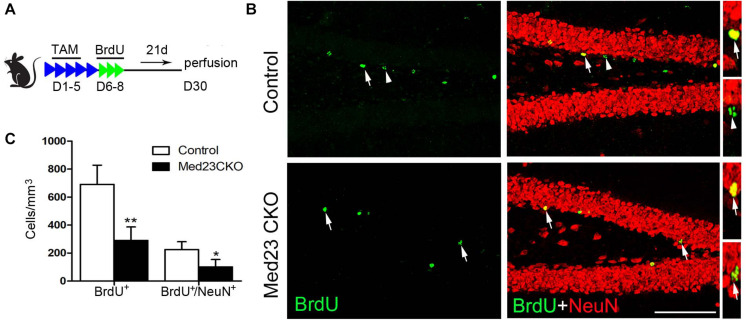
Mature new-born neurons is also reduced in Med23 CKO mice. **(A)** Schematic representation of TAM treatment and BrdU injection protocol for the examination of newborn neuron survival. **(B)** Double immunostaining of BrdU (green) and NeuN (red). Arrows point to BrdU/NeuN-double positive cells and triangles point to cells labeled with BrdU only. Scale bar = 100 μm. **(C)** Quantification data show that the numbers of BrdU-single and BrdU/NeuN-double positive cells are significantly reduced in Med23 CKO mice compared with controls. *N* = 5 in each group. Data are plotted as the mean ± SEM. Student *t*-test, *p < 0.05, **p < 0.01.

In addition, BrdU was administered in 2 month-old mice, and BrdU-labeled newborn neurons were examined 3 weeks later (Figure 5A) by double immunostaining of BrdU with NeuN (Figure 5B). Quantitative analysis revealed that the number of BrdU^+^ cells (Figure 5C) and BrdU^+^/NeuN^+^ neurons (Figure 5C) were significantly decreased in the SGZ of Med23 CKO mice compared with controls. Consistent with the reduction of GFP^+^/NeuN^+^ neurons mentioned above, mature new-born neurons are reduced in the DG of Med23 CKO mice, which is likely to be a consequence of the reduction of neuroblasts and immature neurons. In support of this, TUNEL staining and cleaved caspse3 immunostaining did not detect positive signals in the DG of both control and Med23 CKO.

### Defective Dendritic Arborization of Newborn Neurons in Med23 CKO Mice

**FIGURE 6:**
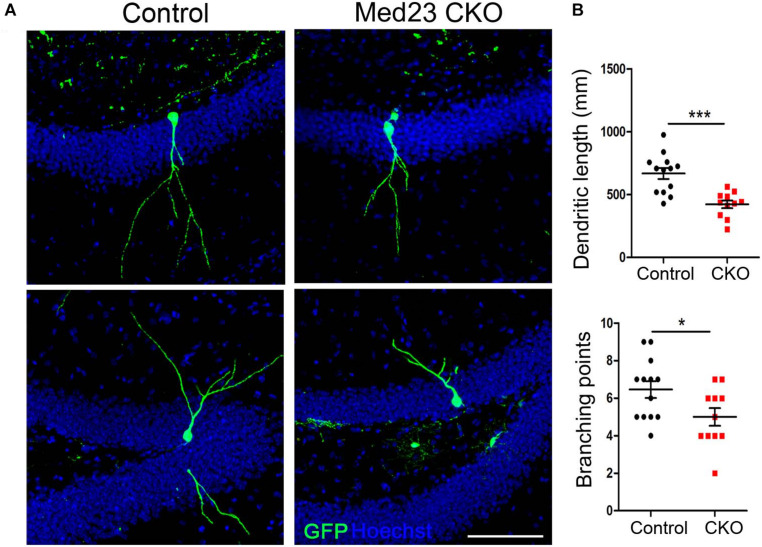
Aberrant dendritic morphogenesis of newborn neurons in Med23 CKO mice. GFP-encoding retrovirus was injected into the dentate gyrus and analyzed 3 weeks later. **(A)** Representative confocal images showing dendrites of newborn neurons in the SGZ of Med23 CKO and control mice. Hoechst (blue) was used as counterstaining. Scale bar = 100 μm. **(B)** Quantification of the total dendritic length and total branching points of GFP^+^ newborn neurons. A total of 13 neurons from 3 control and 11 neurons from 3 Med23CKO mice were included. Data are plotted as the mean ± SEM. Student *t*-test, *p < 0.05, ***p < 0.001.

We next examined dendritic morphogenesis of the surviving newborn neurons in Med23 CKO mice. To this end, we stereotactically injected GFP-expressing retrovirus infecting dividing cells into the DG, and examined dendritic tree of newborn neurons 3 weeks later. Sparse labeling of newborn neurons allowed us to clearly visualize and quantify the dendritic arborization of individual newborn neurons. Dendritic length of GFP-labeled neurons was reduced (Figure 6A) and dendrites were less branched in Med23 CKO mice relative to controls (Figure 6B). These findings are consistent with DCX immunostainings in which we observed many fewer dendrites in DCX^+^ neuroblasts in the SGZ of Med23 CKO mice compared with controls (Figure 4C). Thus, the loss of Med23 leads to aberrant dendritic morphogenesis of newborn neurons in the SGZ.

### Defective Spatial Memory and Contextual Fear Memory in Med23 CKO Mice

**FIGURE 7:**
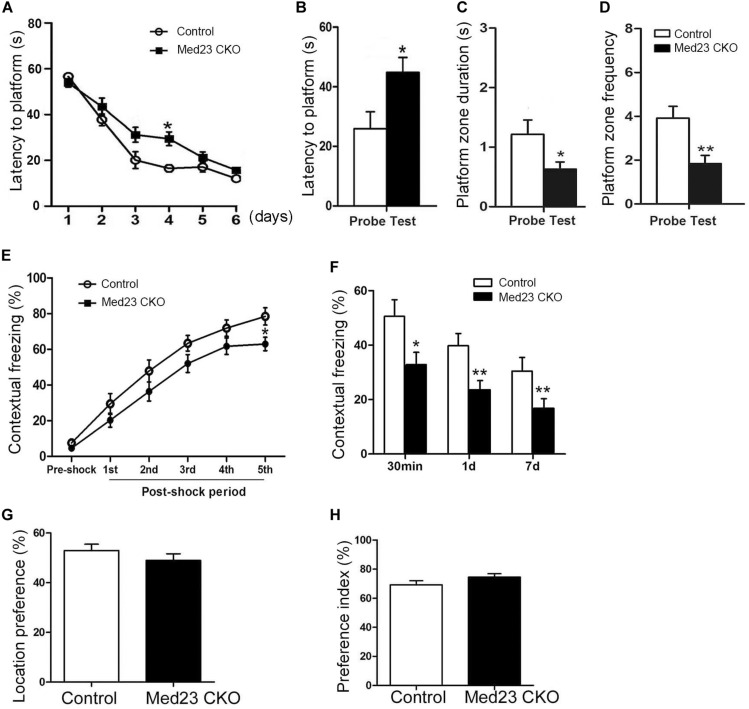
Defective spatial memory and contextual fear memory in Med23 CKO mice. **(A–D)** Morris water maze test. Learning curve of Med23 CKO mice is similar to that of control mice in the probe training except at day 4 **(A)**, but in the probe test Med23 CKO mice show longer latency to platform **(B)**, less time spent in the platform zone **(C)**, and reduced frequency of crossing the platform **(D)** compared with controls. **(E,F)** Contextual fear memory test. Foot shocks provoke a significant decrease in freezing behavior at 5th shock in Med23 CKO mice during conditioning period **(E)**, and Med23 CKO mice also display reduced freezing behavior in the retrieval of contextual fear memory at all time points post conditioning compared with control mice **(F)**. **(G,H)** In the novel object recognition test, no differences in location index **(G)** and recognition index **(H)** are detected between Med23 CKO and control mice. *N* = 12 mice in control and 13 mice in Med23 CKO groups **(A–F)**; *n* = 7 mice in control and 8 in Med23 CKO groups **(G,H)**. Data are plotted as the mean ± SEM. Student *t*-test, *p < 0.05, **p < 0.01.

The Morris water maze was used to investigate hippocampus-dependent spatial learning and memory. Spontaneous locomotor activity was examined in the open field test first, and no differences were observed in traveled distance or velocity between the two groups (data not shown). In the training phase of Morris water maze, the learning curves shown by escape latencies in finding the platform over a 6-day training was comparable, although the latency was significantly increased in Med23 CKO mice as compared with controls on day 4 (Figure 7A). One day after the 6-day training, we examined the retrieval of spatial memory in the absence of the platform. Med23 CKO mice required significantly more time to locate the platform (Figure 7B), spent less time in the platform zone (Figure 7C) and had a reduced frequency of crossing the platform in the probe test compared with control mice (Figure 7D). It should be noted that Med 23 CKO and control mice showed similar velocity during the training and probe test. Thus, a deficiency in spatial memory was observed in Med23 CKO mice.

Adult hippocampal neurogenesis has been shown to be implicated in contextual fear memory (Antunes and Biala, 2012). To explore whether the acquisition and retrieval of the fear memory was changed in Med23 CKO mice, we conducted a contextual fear test as described in our previous studies (Dai et al., 2008; Song et al., 2016). Contextual fear conditioning was tested using foot shocks, and the freezing behavior was measured as the percentage of time spent in freezing during the conditioning and retrieval periods. Interestingly, the freezing behavior was not different during the initial four post-shock intervals, but significantly decreased after the final foot shock in Med23 CKO mice relative to controls (Figure 7E), suggesting a mild impairment in the acquisition of contextual fear memory. When placed back into the conditioned surrounding in the absence of foot shocks, Med23 CKO mice showed less freezing behavior at 30 min, day 1 and day 7 (Figure 7F) post freezing conditioning compared with control mice. The learning and retrieval of contextual fear memory is defective in Med23 CKO mice.

As the above results reflect the possibility that spatial memory and contextual fear memory are impaired in the absence of Med23, we then performed a novel object recognition test to evaluate object recognition memory between two groups. Firstly, we identified no differences in the location index between Med23 CKO and control mice (Figure 7G). We then measured cognitive function using a preference index as described previously (Wang et al., 2007). Both mice showed a preference to the novel object, and no differences in preference index were observed between Med23 CKO and control mice (Figure 7H), suggesting that Med23-deficient may not affect recognitive memory.

### Transcript Profiling of Med23-Deficient NSCs

**FIGURE 8:**
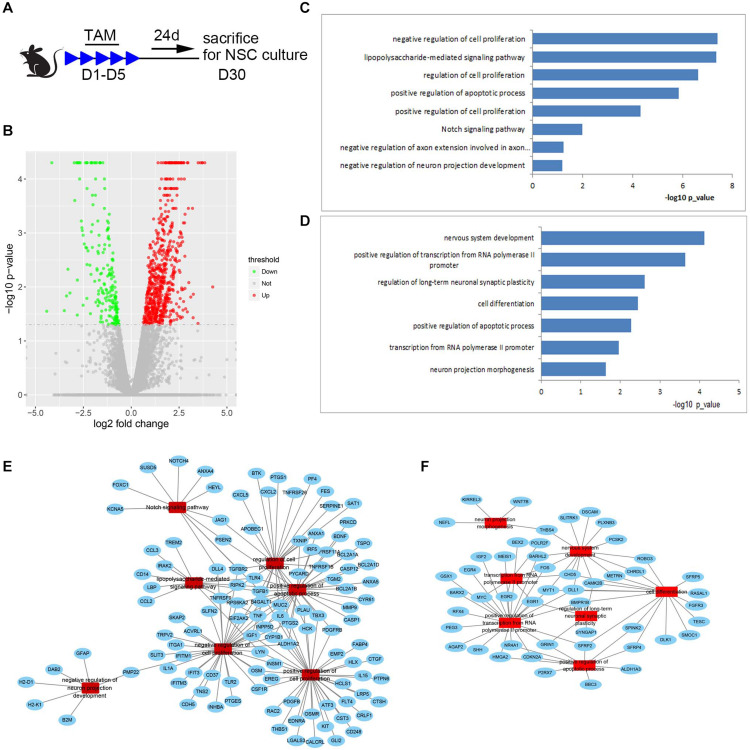
Transcript profiling of Med23-deficient hippocampal NSCs. **(A)** Schematic representation of TAM treatment and sample collection schedule. **(B)** Volcano plots depict gene expression changes between Med23 CKO and control NSCs. Significantly differential transcripts are highlighted in color and totaled in each direction (FDR < 0.05). The cultured neurospheres prepared from individual mouse brain were collected as one sample for RNAseq analysis, and *n* = 3 in each group. **(C,D)** Gene ontology (GO) analysis of the significantly upregulated **(C)** and downregulated **(D)** genes between Med23 CKO and control NSCs. **(E,F)** Protein-protein interaction networks among upregulated **(E)** and downregulated **(F)** genes in Med23 CKO NSCs.

To explore possible cues for future exploring mechanisms underlying the increased number of NSCs and reduced survival of newborn neurons in the SGZ of Med23-deficient mice, we performed transcriptome profiling and analyzed global gene expression patterns of primary neurospheres from Med23 CKO and control mice (Figure 8A, GSE152113). We detected genes that were upregulated (667 genes) or downregulated (196 genes) in Med23-deficient NSCs relative to controls (−log10 *p* < 0.05, Figure 8B). GO analysis revealed that the upregulated genes show an enrichment for cell proliferation (e.g., SAT1, TNF, IGF1, CD37, and EREG), Notch signaling pathway (e.g., DLL4, HEYL, PSEN2, TGFBR2, NOTCH4, and FOXC1) and apoptotic processes (e.g., TSPO, TNF, CYP1B1, PTGS2, MMP9, TLR4, TGFB1, PSEN2, and CASP1) (Figure 8C). Downregulated genes were observed to be enriched for multiple processes important for nervous system development (e.g., SFRP2, SFRP4, SFRP5, EGR1, EGR2, EGR4, DLL1, MYT1, SLITRK1, FOS, FGFR3, SHH, CDKN2A, Wnt7B, and Wnt8B), positive regulation of transcription from Pol II promoter (EGR1, EGR2, RFX4, EGR4, GRIN1, GSX1, BARHL2, NR4A1, BEX2, IGF2, DLL1, HMGA2, MYT1, MEIS1, SHH, FOS, CDKN2A, BARX2, SFRP2, BMPR1B, MYC, AGAP2, andPEG3), and transcription from Pol II (EGR1, FOS, POLR2F, EGR2, MYC, and CHD5) in Med23-deficient NSCs (Figure 8D). Furthermore, analysis of protein-protein interaction networks among upregulated and downregulated genes indicated dysregulations in the regulatory networks involved in cell proliferation, apoptotic processes and neural development in Med23-deficient NSCs (Figures 8E,F). Together, gene regulatory networks particularly in cell proliferation, apoptotic processes and neural development are disturbed in the absence of Med23 in adult hippocampal NSCs.

## Discussion

Whilst several physiological functions have been attributed to Med23, its role in the central nervous system has not been examined *in vivo*. In this study, we focused on adult hippocampal neurogenesis in order to explore the potential role of Med23 in the adult brain as the biological processes are well documented with multiple and reliable study tools. By crossing with Nestin-Cre^ER^ mice, Med23 in hippocampal NSCs could be inactivated by administration of tamoxifen in adult Med23 CKO mice. Conditional inactivation of Med23 in NSCs showed that the loss of this protein leads to an increase of proliferating NSCs but decreases of neuroblasts and new-born neurons in the hippocampus.

The deletion of Med23 with the help of Nestin-Cre^ER^ causes an increase of BrdU^+^, Ki67^+^, and MCM2^+^ cells with no changes in GFAP^+^/Sox2^+^ and Tbr2^+^ populations, suggesting that active NSCs is selectively affected in the population of proliferating cells in the SGZ. However, the populations of neuroblasts and immature new-born neurons shown by DCX^+^ and NeuroD^+^ cells in Med23 CKO mice and YFP^+^ cells in Med23 CKO:Rosa26-YFP mice are significantly reduced. According to the data from single-cell transcriptomics (Llorens-Bobadilla et al., 2015), Med23 is expressed in all cell types with higher levels in active NSCs and neuroblasts in the hippocampal neurogenic niche. Thus, active NSCs and immature new-born neurons require Med23 to maintain its normal biological functions in the SGZ.

In the exploration of possible cellular events leading to the increase of active NSCs, we found that the cell cycle length of proliferating cells in the SGZ is shortened as a result of reduced S-phase length. However, this does not rule out additional possibilities, such as increased cell cycle entry of quiescent NSCs or decreased cell cycle exit of proliferating NSCs toward a quiescent state or differentiation. The methods employed here is originally used in studying the cell cycle of NSCs in the ventricular zone of developing cerebral cortex where homogenous NSCs are located (Chenn and Walsh, 2002), and it was used in addressing the same question in the SGZ later (Qu et al., 2013). However, the progenitors are intermingled with NSCs in the SGZ, and they may have a different cell cycle length, which therefore contributes to the cell cycle phenotypes observed in the SGZ of Med23-CKO mice. This should be clarified in further study.

One of our previous studies has shown that Med23-deficient murine embryonic stem cells show enhanced neural differentiation through modulating BMP signaling (Zhu et al., 2015). In the present study, we show that Med23 is also required for the proliferation of adult hippocampal NSCs and the differentiation of neuroblasts and immature new-born neurons. To our knowledge, this is the first report exploring the role of Med family in the nervous system. As mentioned above, the mammalian Mediator complex is essential to the basal transcription machinery by forming a link between transcription factors and Pol II, whereby it helps regulating the transcription of a large number of protein-coding genes. Consistently, our RNA-seq data show that 667 genes are up- and 196 genes are downregulated in Med23-deficient NSCs. The downregulated portion shows an enrichment for genes involved in Pol II-mediated transcription, multiple processes involved in nervous system development and cell differentiation. In the down-regulated genes of Med23-deficient NSCs, the expression of early response genes (such as Egr1, Egr2, Egr4) that are normally induced by serum growth factor (such as SFRP) activation, are greatly attenuated. These results are consistent with our previous study that Egr1 is the most severely affected by loss of MED23 (Wang et al., 2005). However, Med23-deficient embryonic stem cells display enhanced neural differentiation via BMP signaling (Sun et al., 2014), which is not significantly altered in Med23-deficient hippocampal NSCs.

As mentioned above, Med23 is highly expressed in active NSCs and neuroblasts and the cellular phenotypes are present in the two type of cells suggesting possible cell-autonomous role of Med23 in regulating adult hippocampal neurogenesis. According to the transcript profiling data, Notch pathway is upregulated in the absence of Med23. Notch pathway is required for the maintenance of proliferation capability of NSCs and prevent neuronal differentiation (Louvi and Artavanis-Tsakonas, 2006), and the upregulation of Notch signaling pathway may contribute to the enhanced proliferation of hippocampal NSCs in the absence of Med23. However, Nestin-driven Cre expression is also present in the endothelial cells, and Dll4 and Notch4 are mostly expressed by the endothelial cells (Swift and Weinstein, 2009). This raises a question of if Notch signaling functions in Med23-involved adult neurogenesis in a non-cell-autonomous way. In addition, the upregulated genes involved in cell proliferation (e.g., SAT1, TNF, IGF1, CD37, and EREG) may be implicated in the phenotypes of abnormal neurogenesis of Med23 CKO mice as well. Further studies are needed to explore key downstream effectors that are involved in the Med23-associated transcriptional machinery and regulates adult hippocampal neurogenesis in the mouse brain.

The present findings further demonstrate that Med23 is a factor involved in the regulation of adult hippocampal neurogenesis and its associated role in mouse behavior. As mentioned above, missense mutations in Med23 have been reported in families with autosomal recessive intellectual disability. The mutation (R617Q) leads to a defective response of JUN and FOS immediate early genes to serum mitogens by altering the interaction between enhancer-bound transcription factors (TCF4 and ELK1) and Med23 (Hashimoto et al., 2011; Trehan et al., 2015). Considering the important roles of immediate early genes in brain development, plasticity, and memory formation (Herdegen and Leah, 1998; Tischmeyer and Grimm, 1999; Sanyal et al., 2002; Alberini and Kandel, 2014; Minatohara et al., 2016), it would be interesting to examine possible roles of Med23 in embryonic brain development and in other aspects of adult brain functions. Med23 CKO mice display an impaired performance in the spatial memory, which is consistent with the known functions of adult hippocampal neurogenesis (Ming and Song, 2011; Zhang et al., 2013, 2014), and may be one of factors contributing to the intellectual disability associated with the missense mutation of Med23 (Hashimoto et al., 2011; Trehan et al., 2015). It is well known that animals will learn to link the context itself (the training box) with the foot shocks in contextual fear conditioning, and there is nowadays a consensus that the hippocampus is one of key brain regions for this function (Izquierdo et al., 2016). The Med23 CKO mice show decreased freezing behavior after the last foot shock and during the retrieval period, suggesting impaired fear learning and memory, to which the impaired hippocampal neurogenesis may contribute. In addition, the performance in the object recognition test is normal in Med23 CKO mice. The object recognition can be a different type of memory, which may requires multiple brain regions rather than just the hippocampus.

## Conclusion

In summary, we demonstrate that Med23 plays an important role in multiple steps of adult hippocampal neurogenesis including NSC self-renewal and cell cycle progression, as well as spatial and fear memory. These data provide a strong basis for future investigations about possible roles of Med23 in central nervous system development and function.

## Data Availability Statement

The raw data presented in this study can be found in the GEO, accession number GSE152113.

## Ethics Statement

The animal study was reviewed and approved by the Animal Care and Use Committees of Tongji University School of Medicine, China.

## Author Contributions

G-YC carried out experiments and analyzed the data. SZ, C-HL, and GW helped with RNA-sequencing experiments. Y-ZW helped with TUNEL staining and WB. C-CQ carried out *in situ* hybridization. J-YC helped with mouse work. GW, C-JS, and Y-QD conceived and planned the project. G-YC and Y-QD wrote the manuscript. All authors contributed to the article and approved the submitted version.

## Conflict of Interest

The authors declare that the research was conducted in the absence of any commercial or financial relationships that could be construed as a potential conflict of interest.

## Footnotes

1 https://david.ncifcrf.gov
